# Redetermination of the solvent-free crystal structure of l-proline

**DOI:** 10.1107/S2056989018009490

**Published:** 2018-07-10

**Authors:** Jonas J. Koenig, Jörg-M. Neudörfl, Anne Hansen, Martin Breugst

**Affiliations:** a Department für Chemie, Universität zu Köln, Greinstrasse 4, 50939 Köln, Germany

**Keywords:** crystal structure, l-proline, amino acid

## Abstract

The title compound, (*S*)-pyrrolidine-2-carb­oxy­lic acid (C_5_H_9_NO_2_), commonly known as l-proline, crystallized without the inclusion of any solvent or water mol­ecules through the slow diffusion of diethyl ether into a saturated solution of l-proline in ethanol. l-Proline crystallized in its zwitterionic form and the mol­ecules are linked *via* N—H⋯O hydrogen bonds, resulting in a two-dimensional network. In comparison to the only other publication of a single-crystal structure of l-proline without inclusions [Kayushina & Vainshtein (1965 ▸). Kristallografiya, **10**, 833–844], the *R*_1_ value is significantly improved (0.039 *versus* 0.169) and thus, our data provides higher precision structural information.

## Abstract 2

l-proline crystallized, in its zwitterionic form, without the inclusion of any solvent or water mol­ecules through the slow diffusion of diethyl ether into a saturated solution of l-proline in ethanol. In the crystal, the mol­ecules are linked *via* N—H⋯O hydrogen bonds, resulting in a two-dimensional network.

## Chemical context   

There are 20 proteinogenic amino acids that form the basis of life. Like most amino acids, l-proline predominantely exists in the zwitterionic form (Boldyreva, 2008 ▸; Görbitz, 2015 ▸). Among those proteinogenic amino acids, l-proline is the only compound featuring a secondary amine that can have a significant influence on the structure of proteins and peptides. For example, l-proline is responsible for the secondary structure of collagen (Hutton *et al.*, 1966 ▸) and often acts as a structural disruptor, which leads to structural changes from helical to coil (Tompa, 2002 ▸). Another remarkable influence of the secondary amine can be derived from the hydrogen-bonding pattern in the solid state. Amino acids with primary amino groups commonly form bilayers incorporating two anti­parallel hydrogen-bonded sheets. In contrast, the secondary amino groups in l-proline and its derivatives usually form single-sheet layers, where the amino groups point in the same direction (Görbitz, 2015 ▸). Similar conclusions were also drawn relying on powder diffraction data (Seijas *et al.*, 2010 ▸). Based on the comparison of 40 different amino acids featuring an endocyclic nitro­gen atom, Görbitz concluded that small changes in the mol­ecular composition can cause a significant change in the hydrogen-bonding pattern (Görbitz, 2015 ▸).

Within the last decade, l-proline has also attracted significant attention in the field of organic chemistry as an organocatalyst. Following earlier reports on the application of l-proline in the Hajos–Parrish–Eder–Sauer–Wiechert reaction (Eder *et al.*, 1971 ▸; Hajos & Parrish, 1974 ▸), l-proline was re-discovered as an excellent catalyst for asymmetric aldol reactions (List *et al.* 2000 ▸; Feng *et al.*, 2015 ▸). Today, proline and various derivatives are frequently used catalysts that are routinely employed for many different transformations including aldol, Mannich, Diels–Alder or epoxidation reactions (Mukherjee *et al.*, 2007 ▸). 
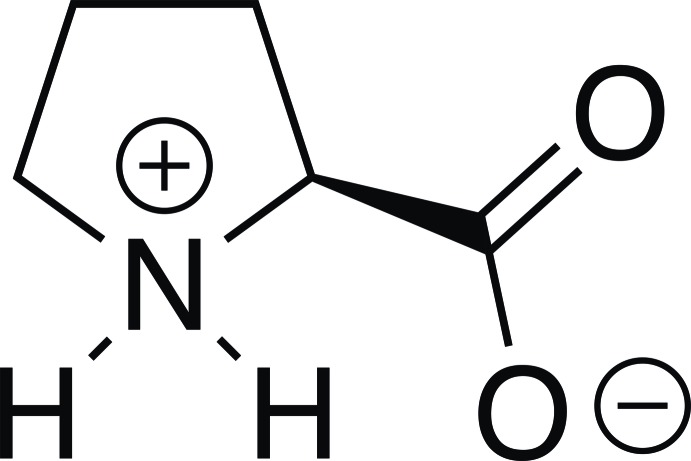


So far, crystal structures with *R*_1_ values of less than 0.10 have been published for 19 of the 20 proteinogenic amino acids (Görbitz, 2015 ▸). However, for l-proline, the only available crystal structure without inclusions dates from 1965 and features a significantly worse *R*_1_ value of 0.169 (Kayushina & Vainshtein, 1965 ▸). To overcome this limitation for the last proteinogenic amio acid, we recently succeeded in determining the crystal structure of l-proline without any inclusions with significantly improved *R*_1_ values.

## Structural commentary   

**Figure 1:**
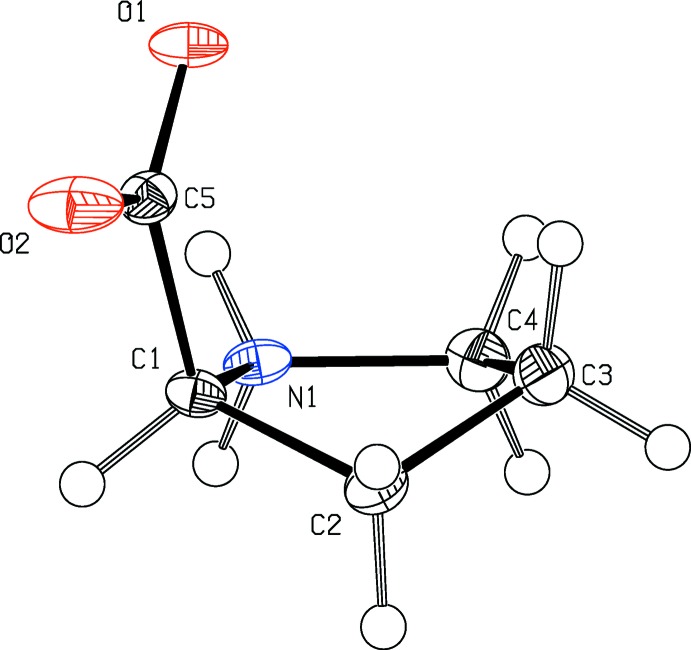
The mol­ecular structure of the title compound l-proline. Displacement ellipsoids are drawn at the 50% probability level.

l-Proline crystallized in its zwitterionic form: the oxygen atoms of the carb­oxy­lic acid (O1 and O2) are deprotonated and accordingly, the amine nitro­gen atom N1 is protonated. The pyrrolidine ring within the title compound adopts a slightly bent envelope conformation with the C2 atom out of the plane (Fig. 1 ▸). Comparing the obtained values with previously reported crystal structures of enanti­omerically pure l- and d-proline, the racemic compound, as well as the co-crystalized structures, only marginal differences can be observed for the distances N1—C1, N1—C4, and C1—C5 as well as for the binding angles C4—N1—C1 and N1—C1—C5. This indicates that the inclusion of solvents and formation of co-crystals does not influence the structural properties of proline significantly.

## Supra­molecular features   

**Figure 2:**
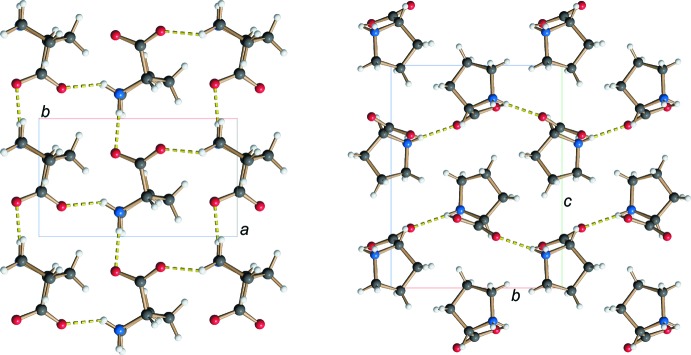
View along the *c* axis (left) and the *a* axis (right) showing that l-proline forms layers through hydrogen bonding between the carb­oxy­lic group O1 respectively O2 and amine N1.

**Figure 3:**
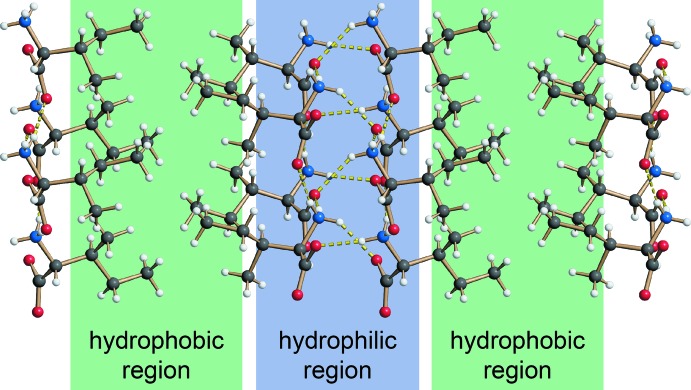
Hydro­philic and hydro­phobic layers in the crystal structure of isoleucin (DAILEU01: Varughese & Srinivasan, 1975 ▸).

As a secondary amine, l-proline carries two hydrogen atoms at the nitro­gen atom N1 in its zwitterionic form. These two hydrogen atoms each inter­act with one of the oxygen atoms of the carb­oxy­lic groups (O1 and O2). The different hydrogen-bond parameters between the proline mol­ecules are shown in Table 1 ▸. As shown in Fig. 2 ▸, these hydrogen bonds result in the formation of a single-sheet architecture within the *ab* plane (also termed sheet *L*1 in Görbitz, 2015 ▸). This structure is addionaly stabilized by hydro­phobic inter­actions between the C—H bonds of the pyrrolidine substructure (see Fig. 2 ▸). In comparison, the hydrogen-bonding pattern of isoleucin (DAILEU01: Varughese & Srinivasan, 1975 ▸) as a typical example of an amino acid with a primary amino group features a double-sheet structure where the hydro­phobic and hydro­philic parts inter­act with each other (Fig. 3 ▸). Therefore, the hydrogen-bonding pattern observed for l-proline once again illustrates why proline is considered to be a structural disruptor in proteins. However, as already pointed out above, small structural changes can have a signifcant influence, as the addition of a hy­droxy group in 3-hy­droxy­proline results in the formation of bands in the supra­molecular structure (HOPROL12: Koetzle *et al.*, 1973 ▸). This again highlights how even small changes such as the addition of a hy­droxy group can change the packing in the crystal structure.

## Database survey   

A survey of the Cambridge Structural Database (CSD, Version 5.39, last update Nov. 2017; Groom *et al.*, 2016 ▸) for the l-proline structure resulted in 16 hits. Only one very early entry refers to the single crystal of the pure l-isomer without any inclusions (PROLIN: Kayushina & Vainshtein, 1965 ▸). However, the determination of this crystal structure was performed in 1965. Nevertheless, Kayushina and Vainshtein could identify the space group as *P*2_1_2_1_2_1_ and determine the cell parameters with *a* = 5.20 Å, *b* = 9.02 Å, *c* = 11.55 Å, which are good, but could be determined with higher precision in this study. Furthermore, the *R*_1_ value has now improved substanti­ally to 0.039. Seijas *et al.* (2010 ▸) investigated the powder diffraction data of enanti­opure l-proline and obtained an *R*_1_ value of 0.089 with similar structural features. They further compared the four pseudopolymorphs of l-proline, l-proline monohydrate, dl-proline and dl-proline monohydrate and concluded that all show a layered packing, which is stabilized by van der Waals inter­actions (PROLIN01: Seijas *et al.*, 2010 ▸).

Besides the single entry for enanti­opure l-proline, one entry refers to enanti­opure l-proline with the inclusion of water (RUWGEV: Janczak & Luger, 1997 ▸), two entries refer to the racemic compound (QANRUT: Myung *et al.*, 2005 ▸; QANRUT01: Hayashi *et al.*, 2006 ▸) and the racemic product with water (DLPROM01: Padmanabhan *et al.*, 1995 ▸; DLPROM02: Flaig *et al.*, 2002 ▸) or chloro­form (WERMIQ: Klussmann *et al.*, 2006 ▸). The enanti­opure l-proline was also crystallized with inclusions of *p*-amino­benzoic acid (CIDBOH: Athimoolam & Natarajan, 2007 ▸), 1,1-di­cyano-2-(4-hy­droxy­phen­yl)ethene (IHUMAZ: Timofeeva *et al.*, 2003 ▸), *S*-bi­naphthol (NISVOA: Periasamy *et al.*, 1997 ▸; NISVOA01: Hu *et al.*, 2012 ▸), *p*-nitro­phenol (QIRNUC: Sowmya *et al.*, 2013 ▸), and thio­urea monohydrate (UFOQEN: Umamaheswari *et al.*, 2012 ▸).

## Synthesis and crystallization   

The crystals were grown from commercially available l-proline (purchased from Carbolution). Crystals suitable for X-ray crystallography were obtained by the slow diffusion of diethyl ether into a saturated solution of l-proline in ethanol. After one night, colourless crystals were obtained and directly investigated *via* single crystal X-ray analysis. ^1^H NMR (500 MHz, DMSO-d_*6*_) *δ* = 1.67–1.83 (2 H, *m*, 3–H), 1.90–2.08 (2 H, *m*, 2–H), 3.02 (1 H, dt, ^2^ *J* = 11.2 Hz and ^3^ *J* = 7.5 Hz, 4–H), 3.22 (1 H, *ddd*, ^2^ *J* = 11.2 Hz, ^3^ *J* = 7.5 Hz, and 5.9 Hz, H–4), 3.65 (1 H, *dd*, ^3^ *J* = 8.7 Hz and 6.5 Hz, 1–H). ^13^C NMR (125 MHz, DMSO-*d_6_*) *δ* = 24.3 (C-3), 29.4 (C-2), 45.7 (C-4), 61.2 (C-1), 169.8 (C-5). [α]D: −85.9° (*c* 1.0, H_2_O) (Lit. Monteiro, 1974 ▸): −85° ± 2° (*c* 1.1, H_2_O), m.p. 486.7–487.2 K (decomposition).

## Refinement details   

Crystal data, data collection and structure refinement details are summarized in Table 2 ▸. All H atoms bonded to carbon were placed with idealized geometry and refined using a riding model with C—H = 0.95 Å, *U*_iso_(H) = 1.2 *U*_eq_(C) for CH, C—H = 0.99 Å *U*_iso_(H) = 1.2*U*_eq_(C) for CH_2_, C—H = 0.98 Å and *U*_iso_(H) = 1.5*U*_eq_(C) for CH_3_. N-bound H atoms were located in a difference electron map and refined isotropically.

## Supplementary Material

Crystal structure: contains datablock(s) global, I. DOI: 10.1107/S2056989018009490/eb2008sup1.cif

Supporting information file. DOI: 10.1107/S2056989018009490/eb2008Isup3.cml

Structure factors: contains datablock(s) I. DOI: 10.1107/S2056989018009490/eb2008Isup3.hkl

CCDC reference: 1852963

Additional supporting information:  crystallographic information; 3D view; checkCIF report

## Figures and Tables

**Table 1:** Hydrogen-bond geometry (Å, °)

*D*—H⋯*A*	*D*—H	H⋯*A*	*D*⋯*A*	*D*—H⋯*A*
N1—H1*A*⋯O2^i^	0.87 (4)	2.01 (4)	2.759 (3)	144 (3)
N1—H1*B*⋯O1^ii^	0.91 (4)	1.82 (4)	2.703 (3)	165 (3)

Symmetry codes: (i) 
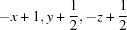
; (ii) 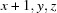.

**Table 2:** Experimental details

Crystal data	
Chemical formula	C_5_H_9_NO_2_
*M*_r_	115.13
Crystal system, space group	Orthorhombic, *P*2_1_2_1_2_1_
Temperature (K)	100
*a*, *b*, *c* (Å)	5.2794 (4), 8.8686 (6), 11.5321 (9)
*V* (Å^3^)	539.94 (7)
*Z*	4
Radiation type	Cu *K*α
μ (mm^−1^)	0.92
Crystal size (mm)	0.40 × 0.10 × 0.08

Data collection	
Diffractometer	Bruker D8 Venture
Absorption correction	Multi-scan (*SADABS*; Bruker, 2012 ▸)
*T*_min_, *T*_max_	0.553, 0.754
No. of measured, independent and observed [*I* > 2σ(*I*)] reflections	4791, 1062, 993
*R*_int_	0.053
(sin θ/λ)_max_ (Å^−1^)	0.618

Refinement	
*R*[*F* ^2^ > 2σ(*F* ^2^)], *wR*(*F* ^2^), *S*	0.036, 0.086, 1.11
No. of reflections	1062
No. of parameters	81
H-atom treatment	H atoms treated by a mixture of independent and constrained refinement
Δρ_max_, Δρ_min_ (e Å^−3^)	0.22, −0.19
Absolute structure	Flack *x* determined using 361 quotients [(*I* ^+^)−(*I* ^−^)]/[(*I* ^+^)+(*I* ^−^)] (Parsons *et al.*, 2013 ▸)
Absolute structure parameter	0.10 (17)

Computer programs: *APEX3* and *SAINT* (Bruker, 2012 ▸), *SHELXT* (Sheldrick, 2015*a* ▸), *SHELXL2014* (Sheldrick, 2015*b* ▸) and *SHELXLE* (Hübschle *et al.*, 2011 ▸), *SCHAKAL99* (Keller & Pierrard, 1999 ▸), *PLATON* (Spek, 2009 ▸) and *publCIF* (Westrip, 2010 ▸).

## supplementary crystallographic information

### Crystal data

**Table:**

C_5_H_9_NO_2_	*D*_x_ = 1.416 Mg m^−^^3^
*M**_r_* = 115.13	Melting point: 486.9 K
Orthorhombic, *P*2_1_2_1_2_1_	Cu *K*α radiation, λ = 1.54178 Å
Hall symbol: P 2ac 2ab	Cell parameters from 4791 reflections
*a* = 5.2794 (4) Å	θ = 6.3–72.3°
*b* = 8.8686 (6) Å	µ = 0.92 mm^−^^1^
*c* = 11.5321 (9) Å	*T* = 100 K
*V* = 539.94 (7) Å^3^	Prism, colourless
*Z* = 4	0.40 × 0.10 × 0.08 mm
*F*(000) = 248	

### Data collection

**Table:**

Bruker D8 Venture diffractometer	993 reflections with *I* > 2σ(*I*)
Radiation source: micro focus	*R*_int_ = 0.053
phi / ω scans	θ_max_ = 72.3°, θ_min_ = 6.3°
Absorption correction: multi-scan (SADABS; Bruker, 2012)	*h* = −6→6
*T*_min_ = 0.553, *T*_max_ = 0.754	*k* = −10→10
4791 measured reflections	*l* = −14→14
1062 independent reflections	

### Refinement

**Table:**

Refinement on *F*^2^	Hydrogen site location: mixed
Least-squares matrix: full	H atoms treated by a mixture of independent and constrained refinement
*R*[*F*^2^ > 2σ(*F*^2^)] = 0.036	*w* = 1/[σ^2^(*F*_o_^2^) + (0.036*P*)^2^ + 0.1571*P*] where *P* = (*F*_o_^2^ + 2*F*_c_^2^)/3
*wR*(*F*^2^) = 0.086	(Δ/σ)_max_ < 0.001
*S* = 1.11	Δρ_max_ = 0.22 e Å^−^^3^
1062 reflections	Δρ_min_ = −0.19 e Å^−^^3^
81 parameters	Absolute structure: Flack *x* determined using 361 quotients [(*I*^+^)-(*I*^-^)]/[(*I*^+^)+(*I*^-^)] (Parsons *et al.*, 2013)
0 restraints	Absolute structure parameter: 0.10 (17)

### Special details

**Table:**

Geometry. All esds (except the esd in the dihedral angle between two l.s. planes) are estimated using the full covariance matrix. The cell esds are taken into account individually in the estimation of esds in distances, angles and torsion angles; correlations between esds in cell parameters are only used when they are defined by crystal symmetry. An approximate (isotropic) treatment of cell esds is used for estimating esds involving l.s. planes.

### Fractional atomic coordinates and isotropic or equivalent isotropic displacement parameters (Å^2^)

**Table:**

	*x*	*y*	*z*	*U*_iso_*/*U*_eq_	
O1	0.2943 (3)	0.61385 (18)	0.31235 (15)	0.0182 (4)	
O2	0.2573 (3)	0.38601 (19)	0.23111 (17)	0.0261 (5)	
N1	0.7901 (4)	0.5949 (2)	0.35050 (17)	0.0150 (4)	
H1A	0.708 (7)	0.673 (4)	0.326 (3)	0.040 (9)*	
H1B	0.952 (7)	0.596 (4)	0.325 (3)	0.034 (9)*	
C1	0.6604 (4)	0.4557 (2)	0.3057 (2)	0.0134 (5)	
H1	0.7482	0.4165	0.2350	0.016*	
C2	0.6869 (4)	0.3449 (2)	0.4064 (2)	0.0171 (5)	
H2A	0.8567	0.2977	0.4071	0.020*	
H2B	0.5563	0.2650	0.4024	0.020*	
C3	0.6479 (5)	0.4456 (3)	0.5127 (2)	0.0186 (5)	
H3A	0.4663	0.4685	0.5246	0.022*	
H3B	0.7164	0.3975	0.5836	0.022*	
C4	0.7967 (5)	0.5875 (3)	0.4816 (2)	0.0191 (5)	
H4A	0.7165	0.6780	0.5160	0.023*	
H4B	0.9733	0.5803	0.5100	0.023*	
C5	0.3804 (4)	0.4883 (3)	0.27998 (19)	0.0150 (5)	

### Atomic displacement parameters (Å^2^)

**Table:**

	*U*^11^	*U*^22^	*U*^33^	*U*^12^	*U*^13^	*U*^23^
O1	0.0086 (7)	0.0153 (8)	0.0307 (9)	0.0011 (7)	0.0002 (7)	−0.0015 (7)
O2	0.0135 (8)	0.0212 (8)	0.0435 (11)	0.0007 (8)	−0.0075 (8)	−0.0108 (8)
N1	0.0083 (9)	0.0136 (9)	0.0230 (10)	0.0000 (8)	−0.0014 (8)	0.0008 (8)
C1	0.0100 (11)	0.0126 (10)	0.0177 (10)	−0.0006 (9)	0.0005 (8)	−0.0019 (9)
C2	0.0167 (12)	0.0143 (10)	0.0202 (12)	−0.0003 (9)	−0.0018 (10)	0.0012 (9)
C3	0.0178 (12)	0.0195 (11)	0.0186 (11)	−0.0004 (10)	0.0011 (9)	0.0015 (9)
C4	0.0175 (11)	0.0196 (11)	0.0201 (12)	−0.0014 (10)	−0.0013 (10)	−0.0036 (9)
C5	0.0115 (10)	0.0167 (11)	0.0168 (10)	−0.0006 (9)	−0.0004 (9)	0.0015 (9)

### Geometric parameters (Å, º)

**Table:**

O1—C5	1.260 (3)	C2—C3	1.531 (3)
O2—C5	1.250 (3)	C2—H2A	0.9900
N1—C1	1.504 (3)	C2—H2B	0.9900
N1—C4	1.514 (3)	C3—C4	1.526 (3)
N1—H1A	0.87 (4)	C3—H3A	0.9900
N1—H1B	0.91 (4)	C3—H3B	0.9900
C1—C2	1.527 (3)	C4—H4A	0.9900
C1—C5	1.535 (3)	C4—H4B	0.9900
C1—H1	1.0000		
			
C1—N1—C4	108.53 (18)	H2A—C2—H2B	109.1
C1—N1—H1A	108 (2)	C4—C3—C2	102.92 (18)
C4—N1—H1A	112 (2)	C4—C3—H3A	111.2
C1—N1—H1B	109 (2)	C2—C3—H3A	111.2
C4—N1—H1B	108 (2)	C4—C3—H3B	111.2
H1A—N1—H1B	111 (3)	C2—C3—H3B	111.2
N1—C1—C2	103.03 (18)	H3A—C3—H3B	109.1
N1—C1—C5	110.50 (18)	N1—C4—C3	105.00 (18)
C2—C1—C5	110.87 (18)	N1—C4—H4A	110.7
N1—C1—H1	110.7	C3—C4—H4A	110.7
C2—C1—H1	110.7	N1—C4—H4B	110.7
C5—C1—H1	110.7	C3—C4—H4B	110.7
C1—C2—C3	102.82 (17)	H4A—C4—H4B	108.8
C1—C2—H2A	111.2	O2—C5—O1	126.0 (2)
C3—C2—H2A	111.2	O2—C5—C1	116.8 (2)
C1—C2—H2B	111.2	O1—C5—C1	117.18 (19)
C3—C2—H2B	111.2		
			
C4—N1—C1—C2	−21.2 (2)	C2—C3—C4—N1	28.2 (2)
C4—N1—C1—C5	97.3 (2)	N1—C1—C5—O2	172.9 (2)
N1—C1—C2—C3	38.5 (2)	C2—C1—C5—O2	−73.5 (3)
C5—C1—C2—C3	−79.7 (2)	N1—C1—C5—O1	−8.7 (3)
C1—C2—C3—C4	−41.5 (2)	C2—C1—C5—O1	104.9 (2)
C1—N1—C4—C3	−4.4 (2)		

### Hydrogen-bond geometry (Å, º)

**Table:**

*D*—H···*A*	*D*—H	H···*A*	*D*···*A*	*D*—H···*A*
N1—H1*A*···O2^i^	0.87 (4)	2.01 (4)	2.759 (3)	144 (3)
N1—H1*B*···O1^ii^	0.91 (4)	1.82 (4)	2.703 (3)	165 (3)

Symmetry codes: (i) −*x*+1, *y*+1/2, −*z*+1/2; (ii) *x*+1, *y*, *z*.
